# Computational Language Assessments of Harmony in Life — Not Satisfaction With Life or Rating Scales — Correlate With Cooperative Behaviors

**DOI:** 10.3389/fpsyg.2021.601679

**Published:** 2021-05-11

**Authors:** Oscar Kjell, Daiva Daukantaitė, Sverker Sikström

**Affiliations:** Department of Psychology, Lund University, Lund, Sweden

**Keywords:** natural language processing (NLP), cooperation, satisfaction with life, computational language assessments, harmony in life

## Abstract

Different types of well-being are likely to be associated with different kinds of behaviors. The first objective of this study was, from a subjective well-being perspective, to examine whether harmony in life and satisfaction with life are related differently to cooperative behaviors depending on individuals’ social value orientation. The second objective was, from a methodological perspective, to examine whether language-based assessments called *computational language assessments* (CLA), which enable respondents to answer with words that are analyzed using natural language processing, demonstrate stronger correlations with cooperation than traditional rating scales. Participants reported their harmony in life, satisfaction with life, and social value orientation before taking part in an online cooperative task. The results show that the CLA of overall harmony in life correlated with cooperation (all participants: *r* = 0.18, *p* < 0.05, *n* = 181) and that this was particularly true for prosocial participants (*r* = 0.35, *p* < 0.001, *n* = 96), whereas rating scales were not correlated (*p* > 0.05). No significant correlations (measured by the CLA or traditional rating scales) were found between satisfaction with life and cooperation. In conclusion, our study reveals an important behavioral difference between different types of subjective well-being. To our knowledge, this is the first study supporting the validity of self-reported CLA over traditional rating scales in relation to actual behaviors.

## Introduction

Different types of well-being are proposed to be associated with different kinds of behaviors (e.g., Ryan and Deci, 2001; Kjell, 2011). Individuals associate the pursuit of harmony in life with *cooperation* and related words (e.g., *together*, *unity*, and *mutual*), whereas the pursuit of satisfaction with life is associated with words relating to self-fulfilment (e.g., *achievement*, *goals*, and *winning*; Kjell et al., 2016). This distinction is also found when having participants describe their *level* (rather than *pursuit*) of harmony in life versus satisfaction with life using open-ended language-based measures, but not when using traditional numeric rating scales (Kjell et al., 2019). The present study allowed individuals to describe their well-being in their own words and had two objectives. The first objective was related to *well-being and cooperation*, i.e., to examine if two cognitive components of subjective well-being – namely, overall harmony in life and overall satisfaction with life – as reported prior to a social dilemma game are related to cooperative behaviors [while also controlling for values in the form of their social value orientation (SVO)]. The second objective was related to the assessment *method*, i.e., to examine whether quantitative open-ended language-based assessments (referred to as *computational language assessments*) more clearly than rating scales distinguish between harmony in life and satisfaction with life in relation to the behavioral outcome of cooperation. These objectives were examined in a social dilemma game where cooperating increased the joint outcome and not cooperating gave one the chance to personally achieve the highest outcome.

### Satisfaction, Harmony, and Cooperation

The definitions of satisfaction with life and harmony in life as well as related empirical research suggest that harmony in life is more related to cooperative behaviors than satisfaction with life. Diener et al. (1985) highlight that satisfaction with life concerns a “cognitive, judgmental process” (p. 71) regarding a person’s evaluation of their life situation as a whole. As such, satisfaction with life is defined as having surroundings and circumstances according to one’s expectations and ideals and in accordance with one’s own criteria (Diener et al., 1985). Harmony in life, on the other hand, relates to being in balance and fitting in with one’s surroundings and circumstances (e.g., see Kjell et al., 2016; Kjell and Diener, 2020). Li (2006) stresses that harmony entails favorable relationships, and Li (2008) points out that “harmony is by its very nature relational. It is through mutual support and mutual dependence that things flourish” (p. 427). Considering the different definitions, harmony in life and satisfaction with life are likely to be associated with different actions and behaviors (e.g., see Kjell, 2011).

Indeed, empirical research demonstrates differences in how individuals view their pursuit of harmony in life and satisfaction with life. In a direct comparison between harmony in life and satisfaction with life, Kjell et al. (2016) revealed that participants describe their pursuit of harmony in life with words relating to interconnectedness with other people (e.g., *peace*, *balance*, *cooperation*, *unity*, *agreement*, *accord*, *concord*, *together*, *friendship*, and *forgiveness*). Meanwhile, the pursuit of satisfaction with life is described with words relating to self-centered (cf. one’s own criteria) mastery (e.g., *money*, *achievement*, *wealth*, *gratification*, *goals*, *work*, *career*, *winning*, *success*, and *job*). Similarly, Kjell et al. (2019) demonstrate that many of these aspects can also be seen in participants’ descriptions of their personal state of harmony in life versus satisfaction with life.

### Cooperation in the Give-Some Dilemma Game

Degree of cooperation in this study was measured in a one-shot give-some dilemma game (GSDG; e.g., see Van Lange and Kuhlman, 1994). In this dilemma game participants are given an amount of money and grouped into pairs. In a simultaneous interaction, they choose to keep their money or give some or all of it to the other person. Participants are informed that any money that is given away doubles in value for the receiver. Hence, keeping the money increases the chance to personally get the highest amount (cf. satisfaction with life), while giving the money to the other person increases the joint outcome (cf. harmony in life). Participants are informed about the other’s decision at the same time. Degree of cooperation is thus operationalised as the amount of money each participant decides to give away. The amount of money participants give is hypothesized to be related to their reported level of harmony in life and satisfaction with life in addition to other factors such as their SVO as discussed next.

### Prosocials and Proselfs

An individual’s SVO is a stable characteristic that predicts the degree of cooperation in social dilemmas (Van Lange and Kuhlman, 1994; VanLange et al., 1997; Balliet et al., 2009), and it is defined as an individual’s preference for a specific resource allocation between others and oneself (McClintock, 1972). Even though individuals can be categorized into several different SVOs, at least three are typically identified: (1) individuals with a *cooperative* SVO who focus on maximizing the joint outcome for self and others, (2) individuals with a *competitive* SVO who focus on maximizing their own outcome relative to others, and (3) individuals with an *individualistic* SVO who focus on maximizing their own outcome with little or no consideration of the outcome for others (VanLange et al., 1997). Individuals categorized with a cooperative SVO are often referred to as *prosocials*, while individuals categorized with an individualistic or competitive SVO are referred to as *proselfs*.

Because SVOs are found to be stable motivations, this distinction has played an important role in research investigating various situational and contextual variables in relation to cooperation. For example, it was demonstrated that inducing guilt (as compared with a neutral state) in participants categorized as proselfs increases cooperation in a prisoner’s dilemma game (Ketelaar and Au, 2003) and a GSDG (de Hooge et al., 2007). In another study using a GSDG, it was demonstrated that inducing fear (as compared with a neutral state) decreases cooperation in prosocials (Nelissen et al., 2007). Further, Kjell and Thompson (2013) compared joy, guilt, and a neutral condition within a prisoner’s dilemma game. That study revealed a significant relationship between cooperation and SVO, but no significant differences in regard to the emotional conditions. It was suggested that cognitive resources and strategies (cf. the cognitive subjective well-being components of harmony in life and satisfaction with life) rather than experimentally induced emotions may have a stronger influence on cooperation. To our knowledge, there are no studies comparing the effect of the cognitive components of subjective well-being (i.e., harmony in life versus satisfaction with life) and their respective relationship to cooperation.

### Open-Ended Computational Language Assessments Versus Numerical Rating Scales

Subjective well-being is typically measured using scales comprising items (e.g., *I am satisfied with life*; Diener et al., 1985) with a closed-ended response format (e.g., ranging from *1* = *Strongly disagree* to *7* = *Strongly agree*). In contrast, Kjell et al. (2019) developed computational language assessments that allow respondents to answer questions about psychological constructs with words that are analyzed using natural language processing. This method enables both *measuring* as well as *describing* the psychological construct under investigation. Importantly it was shown that computational language assessments, as compared to traditional numerical rating scales, discriminate more clearly between harmony in life and satisfaction with life. For example, the numerical rating scales Harmony in Life Scale (HILS; Kjell et al., 2016) and Satisfaction With Life Scale (SWLS; Diener et al., 1985) were strongly correlated, whereas the computational language assessments of harmony in life and satisfaction with life were only moderately correlated. Furthermore, plotting the word responses demonstrated clear differences between words relating to harmony versus satisfaction when plotting according to the computational language assessments. That is, covarying the computational language assessments of harmony in life with satisfaction with life (or vice versa) when plotting significant words yielded a clear independence between the constructs. Interestingly, these differences between harmony and satisfaction were not clear when discriminating between the words using numerical rating scales rather than semantic similarity scales, nor were they clear when covarying the corresponding numerical rating scales. This discriminative property of computational language assessments suggests that they more clearly than numerical rating scales can predict behavioral outcomes that are relevant for one, but not another, psychological construct such as harmony in life and satisfaction with life.

### Objectives and Hypotheses

The study had two objectives. The first objective was to examine if overall harmony in life and overall satisfaction with life reported before a social dilemma game are related to cooperative behaviors (while also controlling for values in the form of their SVO). The hypotheses related to this objective concerned how differently the pre-interaction language-based and numerical measures of harmony in life and satisfaction with life are related to cooperation in the GSDG depending on the individual’s SVO.

H_1_. Level of overall harmony in life correlates positively with cooperation, especially in those categorized as prosocial.

H_2_. Level of overall satisfaction with life correlates negatively with cooperation, especially in those categorized as proself.

The second objective was to examine whether *computational language assessments*, as compared with rating scales, more clearly distinguish between harmony in life and satisfaction with life in regard to cooperation in the GSDG. This is, for example, based on evidence showing that computational language assessments, as compared with numerical rating scales, discriminate more clearly between constructs (Kjell et al., 2019). Therefore, it was hypothesized that:

H_3_. Computational language assessments discern the predictions in H_1_ and H_2_ more strongly than numerical rating scales (i.e., they reveal stronger correlations).

H_4_. The relationships in H_1_ and H_2_ are also discerned using keyword plots based on the computational language assessments. Descriptive words that participants use to describe their overall harmony in life (e.g., *peaceful* and *balance*) are associated with high cooperation, whereas words describing their overall satisfaction with life (e.g., *happy* and *fulfilled*) are associated with low cooperation.

## Materials and Methods

### Participants

Participants were recruited from Amazon’s Mechanical Turk, a website that enables one to pay participants to partake in studies (Paolacci et al., 2010; Mason and Suri, 2012). A total of 200 participants were recruited at once, before starting the analyses. The size of the sample was based on an 80% power to detect a correlation of *r* = 0.2 (alpha level = 0.05, two-sided), which is a correlational size that can be considered theoretically relevant for the investigated hypothesized positive correlation between harmony in life and cooperation. Four participants were removed due to failing to correctly respond to control items (a method that has been shown to increase the statistical power and reliability of datasets; e.g., see Oppenheimer et al., 2009), two were removed for raising suspicion of responding insincerely and not answering the questions independently^1^, and 13 were removed for being suspicious about the authenticity of the interaction in the last feedback question (see section “Material”). The final sample consisted of 181 participants (female = 86; male = 94; other = 1) with a mean age of 34.34 (*SD* = 10.21; range = 19–63) years and a mean of 4.6 (*SD* = 1.7) on the perceived financial situation scale (range 1 = “Our income does not cover our needs, there are great difficulties” to 7 = “Our income covers our needs, and we can save”). Participants mainly came from the United States (United States = 156; India = 20; other countries = 5).

### Material

#### Rating Scales Measures

The HILS (Kjell et al., 2016) consists of five items (e.g., “I am in harmony”) answered on a 7-point scale ranging from 1 = Strongly disagree to 7 = Strongly agree. Cronbach’s alpha in the current study was 0.94 (Mc Donald’s ω total = 0.96).

The SWLS (Diener et al., 1985) comprises five items (e.g., “I am satisfied with my life”) answered on the same scale as the HILS. Cronbach’s alpha in the current study was 0.92 (McDonald’s ω total = 0.95).

The *Triple-Dominance Measure* (TDM; VanLange et al., 1997) was used to assess SVO. The TDM comprises nine items, which each present three distributions of “valuable points” that are differently shared between the respondent and a hypothetical unknown other person. Distributions with equal division of valuable points are categorized as *prosocial*, and distributions where respondents get more than the other are categorized as *proself*. If six or more answers consistently fall within one of the categories, the respondent are classified accordingly.

*Demographic questions* included gender, age, first language, and country of origin as well as perceived financial situation (i.e., “Does the total income of your household allow you to cover your needs?”; answered on a scale ranging from 1 = “Our income does not cover our needs, there are great difficulties” to 7 = “Our income covers our needs, and we can save”).

The *control items “*On this question please answer the alternative ‘neither agree nor disagree”’ and “Answer ‘disagree’ on this question” were included with the numerical rating scales to ensure that the participants had read the questions within the survey. Participants that did not answer these items correctly were removed from the analyses. This kind of method has been demonstrated to ensure high statistical power and reliability (e.g., see Oppenheimer et al., 2009).

#### Word and Text Measures

The *Word-Response Harmony Question* (Kjell et al., 2019) is stated as “Overall in your life, are you in harmony or not?” The *Word-Response Satisfaction Question* (Kjell et al., 2019) reads “Overall in your life, are you satisfied or not?” These word-response questions are presented with the instructions to answer using 10 descriptive words for each question (for full instructions, see Kjell et al., 2019).

*A Feedback Question* asked participants to provide a brief description of their thoughts regarding the GSDG. Three psychology researchers (two with a Ph.D. and one Ph.D. student) not involved in the study, and blind to how the participants responded to other questions, evaluated the answers based on whether they raised any suspicion that the interaction did not involve another person. Participants were removed when at least two out of the three assessors indicated raised suspicion. In total 13 participants were removed (all three assessors agreed on 12 answers and on 1 answer two raters indicated suspicion; only one other answer was indicated as raising suspicion by one assessor, which was thus kept).

The *Affective Norms for English Words* (ANEW; Bradley and Lang, 1999) enabled the construction of language predicted valence scales (see the section on “Natural Language Processing and Statistical Analyses”). These affective norms comprise a large number of words that have been rated by individuals in terms of valence, arousal, and dominance. The valence model used in this study to predict the valence of responses demonstrated a cross-validated Pearson r of 0.73 (*p* < 0.001, *N* = 1025).

#### Intervention

The GSDG (Van Lange and Kuhlman, 1994; de Hooge et al., 2007) involved giving each participant $1.0 and the option to give the money to an interacting partner who simultaneously had the same opportunity. However, the experiment involved a deception in which the “partner” consisted of a computer that randomly responded by either giving $0 or $1. Participants were informed that the amount they decided to give away would double in value for the receiver but that none of the parties in the interaction would know in advance what the other decided to give. The available alternatives to give ranged from $0 to $1, with $0.1 increments. The degree of cooperation was measured as the amount of money the participant decide to give. This was a “one-shot” interaction, meaning that it only took place once.

### Procedure

Participants were informed that the study required English as the first language, that it was voluntary to partake, and that they had the right to withdraw at any time. Further, they were informed that the experiment involved interacting with another person regarding money, and this description was aimed to be as neutral as possible by avoiding more value-laden words such as being cooperative or about winning or losing. Participants were paid $0.5 to complete the study and told that they would keep any money from the interaction task.

After having agreed to partake in the study, participants were informed about how the interaction task (i.e., the GSDG) works and that both parties had to submit their response before they were shown the other’s response. To ensure that the participants had understood the task, they had to answer hypothetical questions correctly before being able to continue (see Supplementary Material Appendix I). Subsequently, participants were presented with the demographic questions, followed by the well-being questions. Participants were randomly assigned to either answer the word-response questions in random order first or the rating scales in random order first.

Before the interaction task started, participants were presented with a message reading, “Searching for another person. Please wait,” and after 16 s another sign popped up reading, “Connecting you with another person.” Participants were then presented with a summary of the instructions of the game and the response alternatives regarding the amount to give to the other person. When they had answered, the participants were presented with the text reading, “Please wait while processing. The other person cannot see your response.” This was followed by the message: “Please wait for the other person to submit their decision.” Subsequently, they were presented with the result of the task (e.g., “The other person decided to give you $0. You gave $0. In total, you get $1 and the other person gets $1.”).

After the interaction, participants answered two questions about their momentary experience of harmony in life and satisfaction with life (which were not analyzed or reported in this study due to its exploratory nature) followed by the TDM. Lastly, before being debriefed, the participants were asked to leave feedback about the interaction. The study took on average 16 min to complete.

### Ethical Considerations

The studies received ethical approval from the Regional Ethical Committee in Lund, Sweden. Prior to participating, all participants received information about the study and were asked for consent to participate. They were informed that participation was anonymous and voluntary and that they could withdraw at any time without having to give a reason. At the end of the study, the participants were given more information about the study and were informed about the deception and why it was important, and they were informed that because of this deception they received the maximum possible amount from the GSDG.

### Natural Language Processing and Statistical Analyses

#### The Semantic Space and Representations

The word data were analyzed with the r-package Text 0.9.0^2^ (Kjell et al., 2021). The words generated in the current study were given their semantic representations (i.e., vectors of numeric values describing each word) from a previously created semantic space (used and described in Kjell et al., 2021). The semantic space was created using latent semantic analyses (Landauer and Dumais, 1997) based on singular values decomposition (Golub and Kahan, 1965) on the co-occurrences of 1.7 × 10^9^ words from the English Google 5-g database. The semantic space includes semantic representations for the 120,000 most frequent English words, in which each word is described in 512 dimensions (for more details, see Kjell et al., 2016).

Word responses were cleaned in accordance to the procedures put forward in Kjell et al. (2019). Words were spelled according to American spelling, and misspelled words were corrected only when the meaning was clear, otherwise they were ignored. Successively repeated words or instances of “NA” or similar were removed. Answers comprising sentences or strings of words rather than one descriptive word in each response box were removed. And words that did not have a semantic representation in the semantic space were returned as missing values.

Because the responses to the word-response questions comprised several words, the semantic representations of the words were added together using the mean of each dimension to create one representative semantic representation for each word-response question. These semantic representations were subsequently used to create *semantic similarity scales*, language predicted *valence*, and the *word plots* as specified below.

#### Semantic Similarity Scales (SSS)

The values that compose the semantic representations can be seen as coordinates in a high-dimensional space, and the closer together the semantic representations of two words/texts are the more semantically similar they are. Hence, the semantic similarity between two words/texts can be represented by the cosine of the angle between the two semantic representations (Landauer and Dumais, 1997). In the current study, we measured the level of a psychological construct by measuring the semantic similarity between responses to the word-response questions and the corresponding word-norms. For example, if a person’s response to the harmony in life question was semantically similar to the harmony in life word-norm, this person was considered to have a high level of overall harmony in life. High unipolar semantic similarity is the semantic similarity to the targeted construct (e.g., harmony in life), low unipolar semantic similarity scales are the opposite of the target constructs (e.g., disharmony in life), and bipolar semantic similarity scales are the low unipolar scale subtracted from the high unipolar scale (e.g., the harmony in life SSS minus the disharmony in life SSS).

#### Language Predicted Scales

The values in the semantic representations can also be used in multiple regressions to create models predicting certain semantic characteristics of a word/text. In the current study, we employed language predicted valence scales. These are based on the ANEW word list where approximately 1,000 words have been rated by individuals in terms of their negative or positive valence. In the multiple regression (*y* = *c*^∗^*x*), the semantic representations (*x*; i.e., vectors) of the words were used to predict the valence (*y*) rated by participants, in which the coefficient (*c*) describes the relationship between the words and the valence. This regression model was applied to the word responses in the current study to estimate their valence (i.e., the regression model was a language predicted valence scale). This model was created using ridge regression (with a penalty grid ranging from 10^–16^ to 10^16^), where cross-validation was used to evaluate the model (for more details, see Kjell et al., 2021).

The SSS and the language predicted scales were used in the correlations to understand their relationship to rating scales and cooperation.

#### Supervised Dimension Projection Plots

Plots were used to visualize words that were statistically significant in relation to the specified categories or dimensions (i.e., axes) under investigation. In the current study words that significantly differed in their semantic representation between responses to the harmony in life versus the satisfaction with life questions were plotted on the *x*-axis, and on the *y*-axis the words were plotted according to the degree of cooperation. Words that statistically significantly differed on a specified dimension, were plotted in color (rather than in gray), and the font size of the word indicated its frequency in the data.

The supervised dimension projection plot compares two groups’ responses to different questions (e.g., harmony in life versus satisfaction with life responses) or low versus high cooperation on a scale using mean split. To achieve this, a semantic representation is first constructed to capture the difference between the two groups, and this semantic representation (point in space) can be seen to form a line through the origo (and is referred to as *the aggregated direction embedding line*). The *aggregated direction embedding* is constructed by taking the mean of all semantic representations in each group and then subtracting the two representations.

Finally, all the individual words in the word responses are “projected” onto the *aggregated direction embedding* line. The projection is achieved by first “anchoring” all of the individual words’ representations in space by subtracting the second group’s aggregated semantic representation from each word’s representation and then using the dot product to project each word’s anchored representation (for more details, see Kjell et al., 2021). To statistically test the words, a dot product null distribution is created by calculating the dot product among randomly selected semantic representations and an *aggregated direction embedding* created from randomly swapping words’ semantic representations from the two groups. Multiple comparisons are corrected using the false discovery rate (FDR) correction.

#### Statistical Analyses

To examine the relationships between variables, Pearson *r* are used when both variables are normally distributed, and Spearman’s *rho* are used when at least one of the variables are not normally distributed. To examine the relationship between two variables whilst controlling for other variables we use partial correlation (e.g., see Kim, 2015).

#### R-Packages

All analyses were carried out in R (R Core Team, 2020) using RStudio (RStudio Team, 2020). Apart from the text package (Kjell et al., 2021), the following packages were used: tidyverse (Henry and Wickham, 2020), Hmisc (Harrell et al., 2020), dplyr (Wickham et al., 2020), ppcor (Kim, 2015), psychometric (Fletcher, 2010), reshape2 (Wickham, 2007), ggplot2 (Wickham, 2016, p. 2), data.table (Dowle and Srinivasan, 2019), lm.beta (Behrendt, 2014), lattice (Sarkar, 2008), effsize (Torchiano, 2020), and WRS2 (Mair and Wilcox, 2019).

## Results

### Descriptive Statistics

Ninety-six participants (53%) were categorized as prosocials, 70 (39%) were categorized as proselfs, and 15 (8%) were uncategorized. On average the participants gave $0.45 (*SD* = 0.41; prosocials: Mean = $0.52, *SD* = 0.41; proselfs: Mean = $0.34, *SD* = 0.39). The cooperation variable exhibited a bimodal, rather than a normal, distribution, and the semantic similarity scales contained some considerable outliers. Because a few participants had, for example, just replied *yes* or *no* to the word-response questions, and because both of these opposing answers yielded outliers of low semantic similarity, outliers with a *z*-score more extreme than ±3.29 were removed for all semantic similarity scales (see Table 1). Table 2 presents correlations among the included well-being measures. The highest correlation was between the HILS and SWLS (*r* = 0.84, *p* < 0.001), whereas the computational language assessments showed lower intercorrelations (e.g., the semantic similarity score of the harmony in life responses and norms with the satisfaction with life responses and norms yielded an *r* of 0.59, *p* < 0.001).

**TABLE 1:** The number of participants excluding missing values, the range, the mean, and the standard deviation before and after outliers were removed for each variable.

**Measure**	***N***	**Range**	**Mean**	***SD***
HILS	181	5–35	26.5	6.34
SWLS	181	5–35	23.9	7.58
H-LPV	180	3.28–7.84	6.07	0.97
S-LPV	178	3.25–7.63	5.98	1.00
H-SSS	180	−0.03–0.72	0.33	0.16
S-SSS	178	0.04–0.72	0.33	0.14
Dh-SSS	179	0.01–0.36	0.16	0.08
Ds-SSS	178	0.03–0.64	0.27	0.10
Ds-SSS no outliers^1^	177	0.03–0.56	0.26	0.09

**N, number of participants excluding missing values; SD, standard deviation. HILS, Harmony in Life Scale; SWLS, Satisfaction with Life Scale; H, harmony; S, satisfaction; Dh, disharmony; Ds, dissatisfaction; LPV, language predicted valence; SSS, Semantic Similarity Scale. ^1^Only the Ds-SSS variable included outliers.**

**TABLE 2:** Pearson correlations among the wellbeing-related measures.

	**1**	**2**	**3**	**4**	**5**	**6**	**7**	**8**	**9**	**10**
(1) HILS										
(2) SWLS	0.84***									
(3) H-LPV	0.67***	0.61***								
(4) S-LPV	0.65***	0.64***	0.72***							
(5) H-SSS	0.45***	0.42***	0.71***	0.60***						
(6) S-SSS	0.48***	0.51***	0.52***	0.76***	0.59***					
(7) Dh-SSS	−0.24**	−0.18*	−0.18*	–0.03	0.06	0.12				
(8) Ds-SSS	−0.54***	−0.50***	−0.51***	−0.54***	−0.35***	–0.11	0.23**			
(9) H-Dh-SSS	0.54***	0.48***	0.73***	0.56***	0.88***	0.47***	−0.43***	−0.42***		
(10) S-Ds-SSS	0.66***	0.67***	0.69***	0.89***	0.65***	0.85***	–0.02	−0.62***	0.60***	

N = 177–181; *p < 0.05, **p < 0.01, ***p < 0.001. HILS, Harmony in Life Scale; SWLS, Satisfaction with Life Scale; H, harmony; S, satisfaction; Dh, disharmony; Ds, dissatisfaction; LPV, language predicted valence; SSS, Semantic Similarity Scale.

### The Well-Being and Cooperation Objective

**FIGURE 1:**
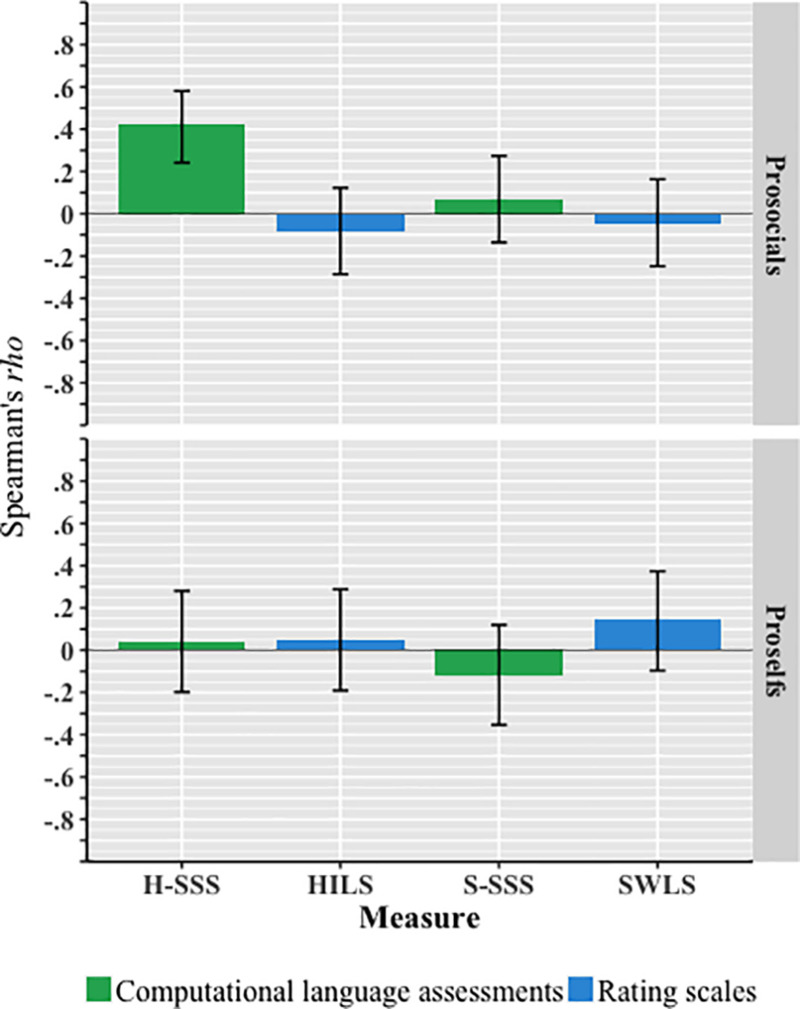
Partial Spearman’s *rho* correlation (with 95% confidence interval) between each well-being measure and cooperation. The data were controlled for the three remaining well-being measures, language predicted valence, perceived financial situation, gender, and age for the various groups. Only H-SSS for prosocials was significant (*r* = 0.41; *p* < 0.001); *n* = 69 prosocials, *n* = 59 proselfs. SSS, Semantic Similarity Scale; H, harmony; S, satisfaction; HILS, Harmony in Life Scale; SWLS, Satisfaction with Life Scale. Note that the *n* differs from Table 3 because partial correlation requires no missing values on all variables.

In accordance with H_1_, the CLA of overall harmony in life (i.e., the SSS between the word-responses of the harmony question and the harmony in life word-norm) was positively correlated with cooperation, and this was strongest in prosocials (*r* = 0.35, *p* < 0.001; see Table 3). However, in contrast to H_1_, this relationship was not found with the HILS. In contrast to H_2_, measures of overall satisfaction with life were not significantly related to cooperation. Figure 1 shows these correlations, where the correlations were controlled for age, gender, perceived financial situation, and all the other well-being-related measures (all presented in Table 3), and only the correlation between the computational language assessment of overall harmony in life and cooperation was significant (*r* = 0.41, *p* < 0.001). It is also worth noting that there is a significant positive correlation between the Disharmony semantic similarity scale and cooperation among proselfs (*r* = 0.39, *p* < 0.001).

**TABLE 3:** Spearman’s *rho* for self-reports and cooperation for the various groups.

**Social value orientation**	**HILS**	**SWLS**	**H-LPV**	**S-LPV**	**H-SSS**	**S-SSS**	**Dh-SSS**	**Ds-SSS**	**H-Dh-SSS**	**S-Ds-SSS**
All (*N* = 181)	0.06	0.09	0.12	0.05	0.18*	0.10	0.27***	0.02	0.05	0.10
Prosocials (*n* = 96)	0.04	0.02	0.21*	0.17	0.35***	0.16	0.17	–0.08	0.25*	0.21*
Proselfs (*n* = 70)	–0.06	–0.07	–0.04	–0.15	–0.09	–0.08	0.39***	0.08	−0.28*	–0.14

*p < 0.05, ***p < 0.001. HILS, Harmony in Life Scale; SWLS, Satisfaction with Life Scale; H, harmony; S, satisfaction; Dh, disharmony; Ds, dissatisfaction; LPV, language predicted valence; SSS, Semantic Similarity Scale.

### The Methodological Objective

In support of H_3_, the distinct prediction of cooperation was shown with computational language assessments but not with numerical rating scales. The computational language assessment of harmony in life also clearly supported the prediction in H_1_, but this was not the case for the HILS. However, in relation to H_2_ there were no significant correlations among the satisfaction with life measures and cooperation (see Figure 1).

### The Computational Language Assessment-Based Plot

**FIGURE 2:**
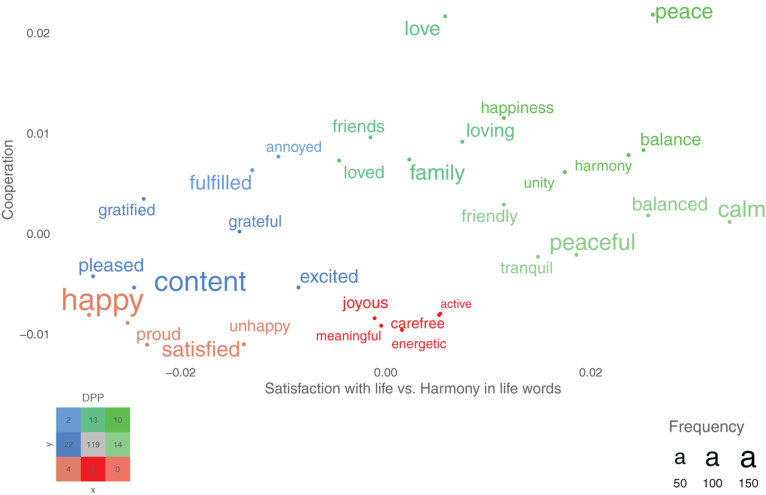
A supervised dimension projection plot of words significantly differing between responses to the satisfaction with life and the harmony in life (*x*-axis) and the level of cooperation (*y*-axis). The colored legend in the lower left corner indicates the color and number of significant words in each part of the figure (for example, there are 10 light green words that are significantly high on both the *x*-axis and *y*-axis). *N* = 180.

Figure 2 shows the statistically significant word responses according to the type of open-ended question (*x*-axis) and to the level of cooperation (*y*-axis). In regard to H_4_, the relationships hypothesized in H_1_ and H_2_ were observed considering that there were more words that were significantly more closely related to high harmony in life that were also significantly related to a high level of cooperation, as compared with high satisfaction with life. That is, 10 words are significant in the right upper corner (see legend; including *peace*, *happiness*, *balance*, *harmony*, and *unity*) whereas there are 0 significant words in the right lower corner. On the other side, there are only 2 words (*fulfilled* and *annoyed*) related to overall satisfaction with life and high cooperation, but 4 words related to satisfaction with life and low cooperation (including *happy*, *proud*, *unhappy*, and *satisfied*).

## Discussion

### Well-Being and Cooperation Objective

We have demonstrated a clear link between subjective well-being and cooperation. Specifically, the computational language assessment of harmony in life yielded a moderately strong significant positive correlation with degree of cooperation in prosocials, while the computational language assessment of overall satisfaction with life did not. This held true even when controlling for all other studied well-being measures (including the traditional numeric rating scales and the predicted valence of the word responses), gender, age, and perceived financial situation.

The word figures further support the importance of harmony in life in relation to cooperation. The statistically different word responses between the harmony in life and satisfaction with life are consistent with previous research; for example, *peaceful* and *calm* are related to harmony in life, and *happy* and *fulfilled* are related to satisfaction with life (Kjell et al., 2019). Importantly, the *Cooperation*-axis further supports that overall harmony in life, but not overall satisfaction with life, is positively related to cooperation, considering that words such as *peace*, *balance*, *harmony*, and *unity* are related to both high harmony in life and cooperation, whereas words such as *happy*, *proud*, and *satisfied* are related to satisfaction with life responses and low levels of cooperation.

Different conditions and situations that support and promote cooperation have been extensively researched (see e.g., Calcott, 2008). Cooperation is a particularly integral part of human society, where human cooperation can be attributed to well-developed *cognitive* resources (Stevens and Hauser, 2004). However, previous research has particularly examined whether certain *emotions* (e.g., Ketelaar and Au, 2003; de Hooge et al., 2007; Nelissen et al., 2007; Kjell and Thompson, 2013) or *positive mood* (Proto et al., 2019) lead to increased cooperation, and less focus has been put on the cognitive component of subjective well-being. To our knowledge, this is the first experiment that tests and demonstrates an association between cooperation and harmony in life measured as the cognitive component of subjective well-being.

Considering the importance of cooperation for societies, we believe that the current results warrant further research interest to deepen the understanding of the link to harmony in life and to satisfaction with life. The results may be seen as particularly important for the subjective well-being literature because there currently is a rather narrow understanding of well-being that predominantly focuses on satisfaction with life. This relates to Kjell’s (2011) concern that a one-sided satisfaction with life focus:

“Appears likely to encourage the individual to put themselves and their expectations first rather than allowing for an adaptive balance of both satisfaction and balance/harmony. Furthermore, measuring satisfaction while neglecting balance/harmony, might crucially relate to the issue that one person’s satisfaction can result in another person’s dissatisfaction.” (p. 260).

Thus, overall, the results give support to the concerns that an overemphasized focus on satisfaction with life can be considered to one-sidedly reflect self-regard and self-centeredness (e.g., see Christopher, 1999; Kjell, 2011), and they suggest that harmony in life is important in complementing satisfaction with life within the subjective well-being approach (see also Kjell et al., 2016).

#### Prosocials and Proselfs

Whereas there was a positive correlation between *harmony* semantic similarity scores and cooperation among prosocials as expected; the results revealed a positive correlation between *disharmony* semantic similarity scores and cooperation among proselfs. That is, among proselfs higher levels of cooperation was related to higher semantic similarity between their harmony in life word-responses and the *disharmony* word norm (i.e., a negative valenced word norm). This finding may perhaps be compared with how inducing proselfs with *guilt* (i.e., a negative valenced emotion) increases their cooperation (Ketelaar and Au, 2003; de Hooge et al., 2007). However, to further understand this relationship among proselfs require further research.

### The Methodological Objective

From a methodological perspective, this study shows that open-ended, computational language assessments of well-being are distinctly related to a theoretically relevant behavioral outcome, whereas standard, closed-ended numerical rating scales are not. As previously discussed, these differences are also discerned in the word figures, where the rating scales method lack an equivalent descriptive analytic method (since rating scales do not allow for descriptive word responses).

To our knowledge this is the first research study that supports the validity of self-reported computational language assessments over traditional rating scales in relation to actual behaviors. Research has, for example, shown that self-reported computational language assessments demonstrate very high convergence with rating scales (Kjell et al., 2021) and that computational language assessments yield higher validity in categorizing external stimuli, including facial expressions (Kjell et al., 2019). There is also evidence that computational language assessments based on individuals’ social media texts (rather than question-based, prompted, self-reports) can predict personality (Schwartz et al., 2013) and are correlated with depression in medical records (Eichstaedt et al., 2018).

Thus, the results presented here add to the research literature demonstrating the validity of computational language assessments. We suggest that future research should attempt to identify the boundary conditions of the computational language assessments (e.g., identifying conditions when ratings scales may have higher validity than computational language assessments and where a combination might be preferred). It would also be valuable to examine respondents’ preferences for the different response formats. For example, which format do respondents prefer in regard to how easy it is to use or how well they can describe their mental states.

### Limitations

The current study has some limitations. It examined only a specific type of cooperation that was constrained to one interaction with an “anonymous” person about money, and participants only received the extreme amounts (i.e., all or nothing). Future studies could also examine harmony versus satisfaction in social dilemmas that, for example, include repeated interactions concerning more aspects than just money. In addition to replicating the current results, future research could examine whether the cooperative link between well-being and cooperation differs in different contexts and situations.

Buhrmester et al. (2011) demonstrated that using Mechanical Turk to collect data produces comparable results as more conventional and standard methods, while also ensuring good generalisability. However, future studies could examine these effects when participants are recruited from other, more social contexts. Further, the analyses statistically controlled for several factors, including perceived financial situation and other well-being measures; however, to further our understanding of the computational language assessments, future studies could control for participants’ current emotional state as well as personality traits. Lastly, the current study did not record the time required to answer the different assessment methods. Whereas Kjell et al. (2019) found that it took longer time for participants to answer the open-ended word format than the rating scales format when describing facial expressions; they also found that only using one rather than ten descriptive words when describing their own mental health produced reliable, although somewhat less accurate, predictions. Future studies could examine how many responses that are necessary while preserving high validity and reliability, how long time each method take to complete and whether respondents prefer one assessment method over the other.

## Conclusion

From a methodological perspective, the results support the validity of computational language assessments, and computational language assessments can distinctly reveal the theoretically relevant behavioral outcome of cooperation within a social dilemma game in relation to subjective well-being, while traditional rating scales cannot. From a well-being perspective, the results reveal a distinct behavioral difference between harmony in life and satisfaction with life, with harmony in life being to a higher degree related to cooperative behavior.

## Data Availability Statement

The datasets generated for this study can be found in online repositories. The names of the repository/repositories and accession number(s) can be found in the article/Supplementary Material. Data will be made available at https://osf.io/bqnar/.

## Ethics Statement

The studies involving human participants were reviewed and approved by Regionala etikprövningsnämnden i Lund. The patients/participants provided their written informed consent to participate in this study.

## Author Contributions

All authors contributed to the study design. OK and SS performed the data collection and the natural language processing analyses. OK, DD, and SS were involved in the other analyses as well as writing up the manuscript. All authors approved the final version of the manuscript for submission.

## Conflict of Interest

OK and SS have co-founded WordDiagnostics, which uses Computational Language Assessments for diagnosing mental health issues. The remaining author declares that the research was conducted in the absence of any commercial or financial relationships that could be construed as a potential conflict of interest.

## Footnotes

1 The two participants were removed for raising suspicion of not completing the study independently. The two participants answered all the word-response questions identically – three of the four questions they answered by repeatedly writing “yes,” and in response to the overall harmony in life question both had, in the same order, answered: “*good*, *marvelous*, *kudos*, *extraordinary*, *elegant*, *resplendent*, *enormous*, *glory*, *stupendous*, *sumptuous.*” On the HILS and the SWLS, one reported a total score of 35 on both scales, and the other a score of 33 on both scales. Further, both reported the same on the demographic questions, gave $1 in the interaction, and answered the third presented alternative on the Triple-Dominance Measure (hence the potential insincerity). They reported different worker IDs but had very similar, overlapping start and submit times. Although keeping these participants in the study lowered the overall correlation between the computational language assessment of overall harmony in life and cooperation, it did not considerably affect the other correlations. Further it did not change the remaining parts of the results as the participants’ SVOs were uncategorized (i.e., not categorized as prosocial or proself).

2 www.r-text.org
