# Supplementary material for: Computational Language Assessments of Harmony in Life — Not Satisfaction With Life or Rating Scales — Correlate With Cooperative Behaviors
Source: Front Psychol. 2021 May 11;12:601679. doi: 10.3389/fpsyg.2021.601679 (PMC8144476; doi:10.3389/fpsyg.2021.601679)
Supplement: Supplementary file 1 [file Table_1.DOCX]

**Supplementary Online Material for:**

**Computational Language Assessments of Harmony in Life**

**— not Satisfaction with Life or Rating Scales —**

**Correlate with Cooperative Behaviours**

**Appendix I. Instructions for the Give Some Dilemma Game**

**Instructions for the interaction**

Later in this study you will take part in an interaction with another person that you will never knowingly meet. In this situation, you and the other person will start with $1 each. Any money you have in the end of the interaction will be paid by us to you as a bonus within Mechanical Turk's bonus system (a maximum of $3).

In the interaction, the two of you will have the options to give money to each other or save your money. You lose the money that you give away, but they double in value for the other person receiving them.

The money doubles accordingly:

$0.0 -->$0.0

$0.1 -->$0.2

$0.2 -->$0.4

$0.3 -->$0.6

$0.4 -->$0.8

$0.5 -->$1.0

$0.6 -->$1.2

$0.7 -->$1.4

$0.8 -->$1.6

$0.9 -->$1.8

$1.0 -->$2.0

The responses will be sent to both of you at the same time, so neither of you will know about the amount that the other person gives in advance. You will only do this interaction once.

To make sure you have understood the options, please answer the questions below.

**If you decide to give $1 and the other person gives you $0 how much would you get in the end:**

**In the example above, how much would the other person get in the end:**

**If you decide to give $1.0 and the other person gives you $1.0 how much would you get in the end:**

**In the example above, how much would the other person get in the end:**

**If you decide to give $0.5 and the other person gives you $0.5 how much would you get in the end:**

**In the example above, how much would the other person get in the end:**

**Instructions at the response stage:**

Please select the amount you want to give to the other person.

- The responses will be sent to both of you at the same time, so neither of you will know the amount the other person gives in advance.
- You will only do this interaction once.
- Both of you get to keep what you have not given away and the amount that is given by the other (where the given money is doubled in value).

**Please select the amount you want to give below.**

$0.0 $0.1 $0.2 $0.3 $0.4 $0.5 $0.6 $0.7 $0.8 $0.9 $1.0
